# Revisiting specific force loss in human permeabilized single skeletal muscle fibers obtained from older individuals

**DOI:** 10.1152/ajpcell.00525.2022

**Published:** 2023-05-22

**Authors:** Michaeljohn Kalakoutis, Ross D. Pollock, Norman R. Lazarus, R. Andrew Atkinson, Marc George, Onur Berber, Roger C. Woledge, Julien Ochala, Stephen D. R. Harridge

**Affiliations:** ^1^Centre for Human and Applied Physiological Sciences, Faculty of Life Sciences & Medicine, King’s College London, London, United Kingdom; ^2^Randall Centre for Cell and Molecular Biophysics, Faculty of Life Sciences & Medicine, King’s College London, London, United Kingdom; ^3^Institut de Pharmacologie et de Biologie Structurale (IPBS), UMR5089, CNRS-Université Toulouse III-Paul Sabatier, Toulouse, France; ^4^Guy’s and St Thomas’ NHS Foundation Trust, London, United Kingdom; ^5^Royal Free London NHS Foundation Trust, London, United Kingdom; ^6^Department of Biomedical Sciences, Faculty of Health and Medical Sciences, University of Copenhagen, Copenhagen, Denmark

**Keywords:** aging, muscle contraction, myosin heavy chain, single muscle fiber, specific force

## Abstract

Specific force (SF) has been shown to be reduced in some but not all studies of human aging using chemically skinned single muscle fibers. This may be due, in part, not only to the health status/physical activity levels of different older cohorts, but also from methodological differences in studying skinned fibers. The aim of the present study was to compare SF in fibers from older hip fracture patients (HFP), healthy master cyclists (MC), and healthy nontrained young adults (YA) using two different activating solutions. Quadriceps muscle samples and 316 fibers were obtained from HFPs (74.6 ± 4 years, *n* = 5), MCs (74.8 ± 1, *n* = 5), and YA (25.5 ± 2, *n* = 6). Fibers were activated (pCa 4.5, 15°C) in solutions containing either 60 mM N-tris(hydroxymethyl)methyl-2-aminoethanesulfonic acid pH buffer (TES) or 20 mM imidazole. SF was determined by normalizing force to fiber cross-sectional area (CSA) assuming either an elliptical or circular shape and to fiber myosin heavy chain content. Activation in TES resulted in significantly higher MHC-I SF in all groups and YA MHC-IIA fibers, irrespective of normalization method. Although there were no differences in SF between the participant groups, the ratio of SF between the TES and imidazole solutions was lower in HFPs compared with YAs (MHC-I *P* < 0.05; MHC-IIA *P* = 0.055). Activating solution composition, as opposed to donor characteristics, had a more notable effect on single fiber SF. However, this two-solution approach revealed an age-related difference in sensitivity in HFPs, which was not shown in MCs. This suggests further novel approaches may be required to probe age/activity-related differences in muscle contractile quality.

**NEW & NOTEWORTHY** Whether specific force (SF) decreases with advancing age in human single skeletal muscle fibers is uncertain. Equivocal published findings may be due to the different physical activity levels of the elderly cohorts studied and/or different chemical solutions used to measure force. We compared single fiber SF between young adults, elderly cyclists, and hip fracture patients (HFP) using two solutions. The solution used significantly affected force and revealed a difference in sensitivity of HFP muscle fibers.

## Graphical abstract

**Figure:**
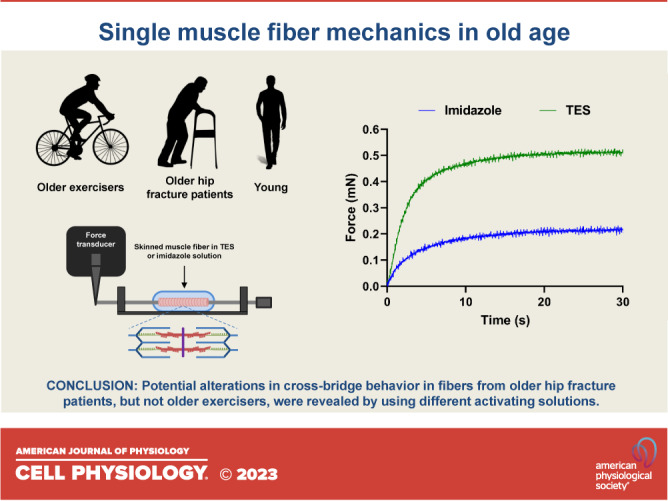

## INTRODUCTION

Age-related weakness has been attributed mainly to a progressive loss of functioning motor units (1, 2) and subsequently skeletal muscle mass, a process termed “sarcopenia” (3). However, the loss of muscle mass underestimates the loss of function in old age (4–6). The observation of a decreased isometric muscle strength normalized to cross-sectional area (CSA), termed specific force (7) (SF), indicates an age-related reduction in muscle contractile quality.

Investigating SF in vivo has multiple challenges from the perspectives of activation (e.g., motor unit recruitment and firing rates), architecture—relating to the determination of physiological cross-sectional area (e.g., accounting for angles of pennation) and joint internal mechanics (8). To overcome many of these limitations, single fiber preparations have been used to study the mechanical properties of permeabilized (skinned) single fibers in vitro. However, even where conditions such as temperature, activation (via pCa) and the chemical concentration of activation solutions can be carefully controlled there remain challenges. SF obtained from different studies and research groups can differ markedly (9) even in fibers obtained from healthy young adults. The reasons for this variability are unclear but in a recent systematic review and meta-analysis (9)_,_ it was concluded that SF heterogeneity may largely be ascribed to differences in the chemical composition of the activating solutions used. For instance, different compounds have been used for the same purpose, such as imidazole or N-tris(hydroxymethyl)methyl-2-aminoethanesulfonic acid pH buffer (TES) for pH buffering in solution.

Studies of single fiber SF in human aging have come from multiple research groups and conclusions are equivocal. Some studies report an age-related reduction in SF (10–15), whereas others report no change (16–23) or an age-related increase (24–28). A further factor that is likely to contribute to this heterogeneity, in addition to methodological differences, is the range of health and physical activity statuses of participants in the different studies (29).

The aim of the present investigation was therefore to address the issue of both different single fiber methodologies and participant/donor heterogeneity to reinvestigate aging and SF in single muscle fibers. We compared skinned fiber SF in two older cohorts, which represent a spectrum of phenotypes: physically active, noncompetitive master cyclists (30) (MC) and hip fracture patients (HFP), with skinned fibers from healthy, young adults (YA). To identify any effect of the activating solution, we measured SF using two solutions used by different research groups, which differed in their choice of pH buffer, using either imidazole (31) or TES (32).

## METHODS

### Ethical Approval

The collection of YA and HFP muscle samples was approved by the Fulham Research Ethics Committee in London (Ethics ID: 12/LO/0250). All subjects provided written informed consent to participate in this investigation. Muscle samples from the MCs had been obtained as part of a previous study approved by the Westminster Ethics Committee in London (Ethics ID: 12/LO/0457) (30). The study ran in accordance with the Declaration of Helsinki (2013) (33).

### Participants

Three mixed sex cohorts were recruited to participate in the present study (Table 1). Six YAs (mean age: 26 ± 2 yr), five MCs (mean age: 75 ± 1 yr), and five HFPs who underwent dynamic hip screw insertion surgery (mean age: 75 ± 4 yr) were recruited.

**Table 1.** Sex and age of male and female participants studied, with the addition of any history of major illness in the hip fracture patient cohort

	Young Adults		Master Cyclists		Hip Fracture Patients		
Subject	Sex	Age	Sex	Age	Sex	Age	History of major illness (other)
1	Female	21	Female	79	Female	78	Breast cancer, lymphoma
2	Female	32	Female	76	Female	59	None
3	Male	22	Male	75	Female	77	Chronic obstructive pulmonary disease (multiple falls in the last year)
4	Male	25	Male	73	Female	82	Hypertension, rheumatoid arthritis, type 2 diabetes
5	Male	29	Male	71	Male	77	Rheumatoid arthritis
6	Male	24					
Mean age		25.5 ± 2		74.8 ± 1		74.6 ± 4	

Values are means ± SE.

YA and MC participants were considered healthy if they met the criteria outlined by Greig et al. (34). Briefly, YAs completed a health questionnaire and were excluded if they were taking any medication or had any musculoskeletal, cardiovascular, or neurological problems. MCs were recruited if they were able to cycle 100 km in under 6.5 h (males) or 60 km in under 5.5 h (females) and had to have done so twice in the 3 wk before testing as part of a previous study (30). MCs completed a health questionnaire and were excluded if they smoked or consumed alcohol excessively, had known hypertension, cardiovascular, musculoskeletal, or neurological conditions, or if they were on any medication. HFPs were identified by members of their healthcare team. Based on a physical health questionnaire, they were considered eligible if they did not suffer from any neuromuscular disease. The HFPs often had comorbidities known to be associated with skeletal muscle SF loss, such as rheumatoid arthritis (35), cancer (36), and chronic obstructive pulmonary disease (COPD) (37).

### Biopsy Procedure

A muscle biopsy was taken from the vastus lateralis (VL) of the YA and MC cohorts using lidocaine as a local anesthetic, followed by a Bergstrom biopsy needle with suction (38). A surgeon collected muscle tissue samples from the upper portion of the VL from HFPs scheduled to undergo dynamic hip screw insertion surgery. All muscle biopsy samples were immediately placed in relaxing solution (Table 2) cooled on ice to 4°C.

**Table 2.** The chemical constituents of solutions used to prepare a muscle biopsy sample for mechanical experiments

Solution	ATP, mM	EGTA, mM	MgCl_2_, mM	Imidazole, mM	KCl, mM	pH
Relaxing	4.05	2	2	10	100	7.0
Skinning	Same as relaxing solution but with 50% vol/vol glycerol.					
Sucrose	Same as relaxing solution but with either 0.5 M, 1 M, 1.5 M, or 2 M of sucrose in solution.					

### Processing of Muscle Samples

While in relaxing solution, the muscle biopsy samples were dissected into ∼100 fiber bundles under a stereo microscope (Zeiss, Stemi 2000-C) with a separate light source (Zeiss SteREO CL 1500 ECO). The fiber bundles were tied to glass capillary tubes using surgical silk (LOOK SP102) and chemically skinned in relaxing solution with 50% vol/vol glycerol at 4°C for 24 h. The chemically skinned fiber bundles were preserved for long-term storage by sequential 30-min incubation periods in a series of four relaxing solutions containing increasing concentrations of sucrose (0.5, 1, 1.5, 2.0 M). Storage in a sucrose cryoprotectant has been found to preserve skinned fiber force production (39). Bundles were snap frozen in isopentane (GPR-RECTAPUR) cooled to −80°C using dry ice, and stored at −80°C.

### Preparation of Muscle Samples for Experiments

On the day of an experiment, a bundle of skinned fibers was incubated in each sucrose solution in reverse order (2 M → 0.5 M) for 30 min, while cooled on ice at 4°C. The bundle was transferred to relaxing solution also cooled on ice, and fine forceps were used to dissect 1–2 mm fiber segments under a stereo microscope. Aluminum t-clips (Photofabrication Ltd, Cambridgeshire, England) were attached to both ends of a fiber, which was transferred to relaxing solution (pCa 9.0) in an experimental chamber on a custom-built skinned fiber test system. The fiber was suspended in solution between two hooks, one of which was secured to a stationary micromanipulator, whereas the other was attached to a force transducer (Kronex, AE-801).

Skinned fibers were viewed from above using a light microscope (Olympus, BXFM-F). Sarcomere length (SL) was set to 2.75 µm using a video sarcomere length camera (900B, Aurora Scientific), and checked using a calibrated eye-piece graticule (Graticules LTD, Tonbridge, Kent) while viewed using a ×40 magnification water immersion objective (Carl Zeiss, ×40, 0.75, N.A.). Fiber condition was assessed at ×40 magnification so that fibers tested did not exhibit signs of damage such as microtears or an unsmooth membrane surface. Fibers were also excluded if they became damaged during the course of an experiment.

**Figure 1.**
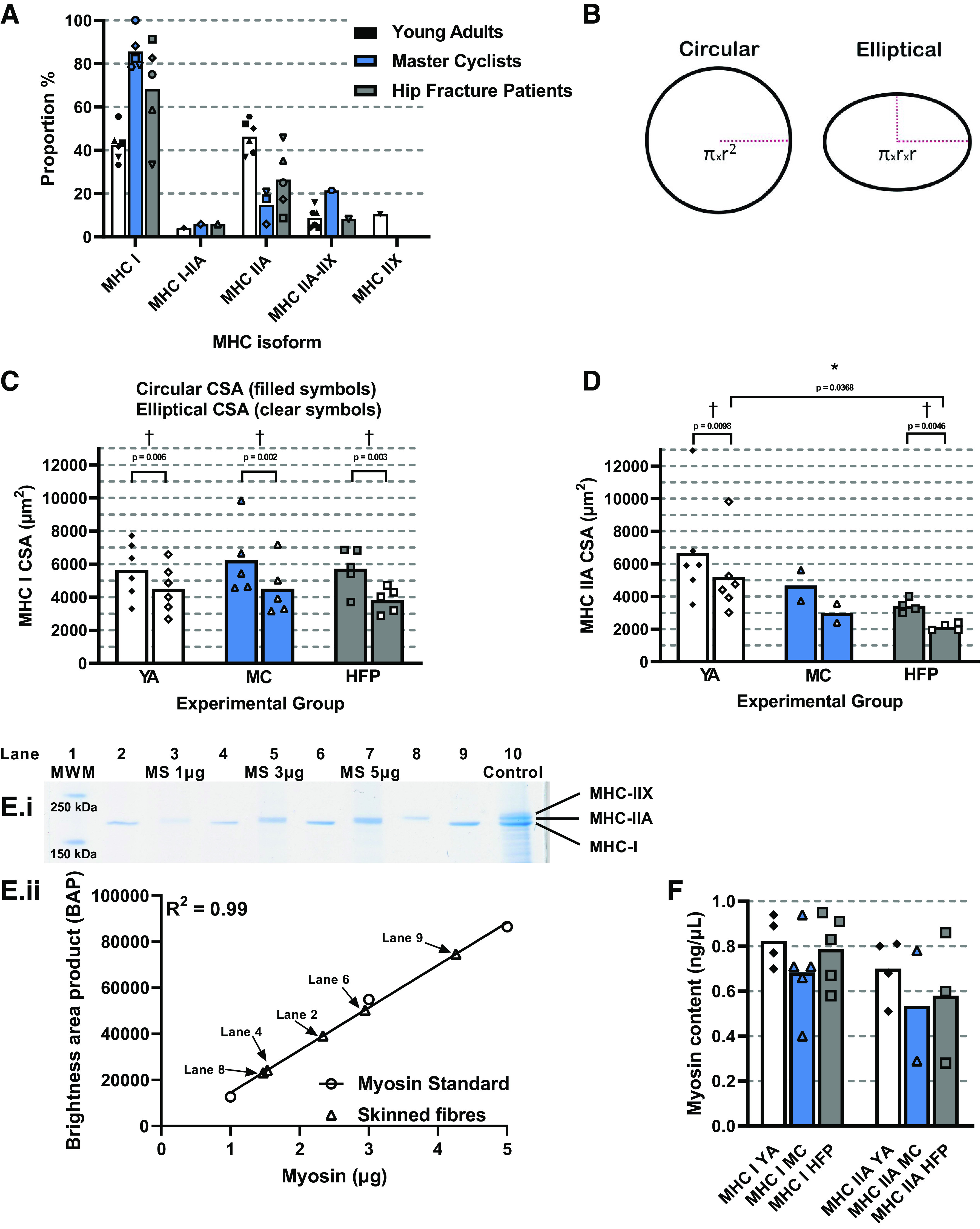
Fiber type distribution, cross-sectional area and MHC content of 316 human skinned fibers studied. *A*: proportion of MHC isoforms expressed. There was a relatively even proportion of 42% (7% SD) and 46% (8% SD) MHC-I and MHC-IIA fibers, respectively, from YAs. MCs exhibited 86% (9% SD) MHC-I fibers and 9% (10% SD) MHC-IIA fibers, and HFPs exhibited 72% (18% SD) MHC-I fibers and 28% (16% SD) MHC-IIA fibers. Data are displayed as means with individual symbols representing individuals from each group. Only one individual from each group had MHC I-IIA fibers and only one MC and HFP had MHC-IIA–IIX fibers. *B*: schematic illustration of CSA calculation assuming either a circular or elliptical cross-sectional shape. *C*: mean MHC-I skinned fiber CSA measurements, YA *n* = 45, MC *n* = 72, HFP *n* = 72; *n* represents the number of individual skeletal muscle fibers studied. Between groups, circular (*P* = 0.8504) and elliptical (*P* = 0.6463) CSA measurements were similar. Within each group, elliptical CSA values were significantly smaller than circular CSA values from the same fibers in YAs (*P* = 0.006), MCs (*P* = 0.002) and HFPs (*P* = 0.003). *D*: mean MHC-IIA skinned fiber CSA measurements, YA *n* = 45, MCs *n* = 7, HFPs *n* = 18. Elliptical CSA values were significantly higher in YAs compared with HFPs (*P* = 0.0368). Within groups, elliptical CSA values were significantly smaller than circular CSA values in YAs (*P* = 0.0098) and HFPs (*P* = 0.0046). *E*: a representative image of an SDS-PAGE gel on which the skinned fiber MHC isoform has been identified and MHC content has been quantified. From left to right, samples are a molecular weight marker (MWM, 150-250 kDa), skinned fiber, MHC standard (MS) or control sample containing all three human MHC isoforms. *F*: mean skinned fiber MHC content from MHC-I (YA *n* = 24, MC *n* = 68, HFP *n* = 67) and -IIA (YA *n* = 20, MC *n* = 5, HFP *n* = 12) fibers from all experimental groups. MHC content (μg) was normalized to the volume of individual skinned fibers from each subject (nL). No statistically significant difference in mean MHC content was observed between groups in MHC-I (*P* = 0.4125) or MHC-IIA (*P* = 0.4944) fibers. MHC content data from two of the six YAs was not collected. Individual data points represent the mean value from an individual subject. MHC-I CSA values were compared between groups using a one-way ANOVA. MHC-IIA CSA values were compared between YA and HFP groups using an unpaired *t* test. Cylindrical vs. elliptical CSA values of both MHC-I and -IIA fibers were compared using paired *t* tests. MHC content values were compared between groups using a one-way ANOVA and an unpaired *t* test for MHC-I and -IIA fibers respectively. *P < 0.05, †*P* < 0.01. CSA, cross-sectional area; HFP, hip fracture patients; MC, master cyclists; MHC, myosin heavy chain; YA, young adults.

Once the SL was set, morphometric measurements were taken. Fiber diameter was measured at three points along the fiber at ×40 magnification. Skinned fiber CSA was calculated assuming a cylindrical cross section, without adjustment for the swelling, which occurs on skinning. Fiber depth was calculated by measuring the displacement of the ×40 objective when focused on the top and bottom of the fiber. This permitted fiber CSA to also be determined assuming an elliptical shape (40). Within groups, elliptical CSA values were significantly (*P* < 0.05) smaller than the mean cylindrical CSA values in YAs (MHC-I *P* = 0.006, MHC-IIA *P* = 0.0098), MCs (MHC-I *P* = 0.002), and HFPs (MHC-I *P* = 0.003, MHC-IIA *P* = 0.0046) for both fiber types (Fig. 1, *C* and *D*). Fiber length was measured using a ×10 objective (BECK, ×10/0.17) and volume was calculated as volume = (π × width × depth × length)/4 and converted to nanoliters. Only the segment between the t-clips was measured and included for further analysis of MHC content. The temperature of the experimental solutions was maintained at 15°C by a water cooler (Neslab RTE-300) and monitored during experiments using a thermocouple (HANNA, HI93530).

### Measurement of Contractile Properties

Skinned fibers were transferred from a relaxing (pCa 9.0) to a preactivating solution for 1 min, then an activating solution (pCa 4.5) where they contracted until a plateau in force signal was reached, before being transferred back into relaxing solution for 2 min before the next contraction sequence began. All experiments were performed at 15°C. P_o_ was calculated as the maximum minus the minimum force value while in activating solution. SF was calculated as the skinned fiber P_o_ normalized to CSA determined assuming a cylindrical cross section and an elliptical cross section without adjustments for swelling. P_o_ measurements were also normalized to MHC content quantified from the same skinned fibers.

Two different activating solutions, “imidazole solution” (31) and “TES solution” (32), were used to measure force from the same skinned fibers. The TES solution was designed using custom written Matlab software, whereas the imidazole solution had been designed using the computer program of Fabiato (41). The chemical constituents of all activating solutions can be compared in Table 3. Free calcium concentration was calculated to be the same (pCa 4.5, 0.03 mM) in both activating solutions and the pH was 7.1. The order in which the activating solutions were used was allocated randomly. P_o_ was measured from each fiber five times: two successive contractions were measured in each activating solution, followed by a final contraction in the initial solution. It was observed that the force produced during the first contraction was lower than the following contraction. Therefore, the first contraction was discounted from data analysis. The reported P_o_ was the mean of two contractions, either the second and fifth or the third and fourth contractions, in a given solution. Data were accepted if P_o_ was consistent within TES and imidazole solution.

**Table 3.** The concentrations of chemicals in the experimental solutions used to study skinned fiber contractile mechanics

Solution	ATP, mM	PCr, mM	EGTA, mM	CaEGTA, mM	MgCl_2_, mM	CaCl_2_, μM	TES, mM	Imidazole, mM	K-Propionate, mM	KCl, mM	Glutathione, mM	Ionic Strength, mM
Relaxing (pCa 9.0)	4.65	14.50	7.00		5.46	17.70		20.00		77.63		200
Preactivating	5.20	19.60	0.50		5.42	16.00		20.00		79.25		200
Imidazole solution (pCa 4.5)	4.70	14.50	7.00			7.00 mM		20.00		62.70		180
TES solution (pCa 4.5)	4.65	20.00	0.17	24.83			60.00		16.00		10.00	200
Imidazole substitution solution (pCa 4.5)	4.65	20.00	0.17	24.83				20.00	18.90		10.00	200
TES dose–response (pCa 4.5)	4.65	20.00	0.17	24.83	7.70		1−100		Enough to adjust ionic strength		10.00	200
Imidazole dose-response (pCa 4.5)	4.65	20.00	0.17	24.83	7.70			1−100	Enough to adjust ionic strength		10.00	200

The pH of all solutions was 7.1. For the imidazole dose-response experiments, the ionic strength needed to be increased to 210 and 220 mM to accommodate concentrations of 80 and 100 mM imidazole, respectively. The slightly different amounts of K-propionate required to adjust ionic strength in each solution are given in Supplemental Table S1.

### SDS-PAGE

Sodium dodecyl sulfate polyacrylamide gel electrophoresis (SDS-PAGE) was used to identify the fiber type of individual skinned fibers based on their myosin heavy chain (MHC) isoform composition. Single skinned fiber segments were placed in standard Laemmli sample buffer (LSB) (42) immediately following force measurements and stored at −80°C until SDS-PAGE experiments. Ten microliters of sample buffer were used per 1 mm of skinned fiber length. The skinned fiber samples were heated at >95°C for 3–5 min before being loaded onto a 0.75 mm thick, 4% stacking gel with an 8% resolving gel, both containing 30% glycerol (43). The upper running buffer contained β-mercaptoethanol (44). Over a 26-h period, a constant current of 4 mA was set until the bromophenyl blue marker had migrated into the separating gel, then increased to 8 mA. Bands were visualized using a commercially available Coomassie blue stain (Novex SimplyBlue safe stain, LC6060) and scanned using a flatbed scanner with high 4800 × 4800 dpi resolution (CanoScan 900 F Mark II, Canon).

The skinned fiber MHC isoform was identified with reference to a control sample (mixed fiber homogenate). Western blotting confirmed the presence of MHC-I and -IIA isoforms in the human control sample tested using the mammalian primary monoclonal antibodies BA-F8 (Immunoglobulin G, IgG) and Sc-71 (IgG), respectively. The other band was identified as MHC-IIX, as it ran in the position typical for that isoform (45) (Supplemental Fig. S7).

The proportion of fibers containing specific MHC isoforms was calculated for each individual, and the mean proportion for each experimental group is illustrated in Fig. 1*A*. One hundred and twenty fibers were studied from YAs, 92 fibers from MCs, and 104 fibers from HFPs. The fiber type proportion was relatively even for YAs, who had 42% (7% SD) MHC-I and 46% (8% SD) MHC-IIA fibers. In contrast, the elderly groups exhibited predominantly MHC-I fibers. 86% (9% SD) of MC fibers were MHC-I compared with just 9% (10% SD) MHC-IIA, whereas 72% (18% SD) of HFP fibers were MHC-I and 28% (16% SD) were MHC-IIA.

### Quantifying MHC Content

The optical density, termed the brightness area product (BAP), of MHC bands from single fibers was quantified using image analysis software (ImageJ). The MHC content was determined within the linear range of three samples (1, 3, or 5 µg) of a commercially available MHC standard (Sigma M7659) loaded onto the same gel. The total MHC content was calculated and normalized to fiber volume, giving a measurement of skinned fiber MHC content per unit of fiber volume. The consistency of the effect of the LSB used to de-nature and extract MHC from skinned fibers was tested as described for Supplemental Fig. S4. MHC content data is available from 14 of the 16 participants studied. The mean values for MHC-I (*P* = 0.4125) and -IIA (*P* = 0.4944) fiber MHC content were similar between all three experimental groups (Fig. 1*F*). MHC content was also similar between MHC-I and -IIA fibers within YA (*P* = 0.2090) and HFP (*P* = 0.221) groups.

### Examining the Effect of the pH Buffer, Major Anion, and Reducing Agent

There were three obvious differences between the imidazole and TES activating solutions: *1*) the choice of pH buffer; *2*) the use of KCl rather than potassium propionate (K-propionate) in the imidazole solution to adjust ionic strength, making chloride the major anion; and *3*) the use of the reducing agent, glutathione, in TES solution. A biopsy from one YA subject was used to test the effect of these chemical differences.

A dose–response curve experiment was carried out to determine the optimal concentration of imidazole or TES pH buffers for force production. A purpose written Matlab program was used to design new versions of TES solution, containing either 20, 40, 60, 80, or 100 mM TES (Table 3, Supplemental Table S1). SF was recorded twice in each solution, which were used in a random order. The data were accepted if a high consistency in force production was observed in each solution. A comparison using the five-contraction protocol described previously was also made between TES solution and an identical solution with 0 mM TES.

To measure the imidazole dose-response curve, solutions were made with 0, 10, 15, 20, 40, 60, 80, or 100 mM of imidazole replacing TES. In 80 or 100 mM solutions of imidazole, the ionic strength was adjusted to 210 and 220 mM, respectively, to accommodate the increased imidazole concentration. Details of the constituents of all solutions are given in Table 3. Dose-response curves were plotted for imidazole concentrations between 0 and 20 mM and between 20 and 100 mM using a 12-contraction protocol. SF was measured twice in each solution, followed by a final pair of contractions in the initial activating solution, which served as an internal control. Imidazole and TES dose-response curves are plotted on Fig. 5*A* for comparative purposes.

To compare the effects of TES and imidazole on P_o_ directly, an optimal concentration of imidazole (20 mM) was substituted into the TES solution in a new solution called “imidazole substitution solution” (Table 3). The logK values for reactions, free ligand and metal ion concentrations are given in Supplemental Figs. S8 and S9. To test the effect of the chloride anion in imidazole solution, a “propionate substitution solution” was made using K-propionate instead of KCl. However, making a version of the imidazole solution, which only differed in the use of K-propionate, was not possible because the software used was written to make TES solution so used different compounds such as CaEGTA rather than CaCl_2_ and EGTA. Therefore, these data must be interpreted with caution. To directly examine the effect of the reducing agent glutathione on P_o_, a chemically identical version of TES solution was made, which excluded glutathione.

### Proton Nuclear Magnetic Resonance Spectroscopy

To identify whether the inclusion of TES or imidazole in the solutions led to the formation of any compounds that might affect the skinned fiber contractile response, proton nuclear magnetic resonance spectroscopy (^1^H NMR) spectra of the solutions were recorded. Samples of 475 µL of each solution were mixed with 25 µL of deuterium oxide (D_2_O) in Eppendorf tubes before being transferred to 5-mm diameter NMR tubes (Wilmad-LabGlass). A “blank” sample containing only the distilled water (H_2_O) used to prepare the experimental solutions, and no other chemicals apart from 25 µL of D_2_O, was also tested to assess the purity of the distilled H_2_O.

1D ^1^H NMR spectra were recorded at 298 K and 700 MHz on a Bruker Avance III 700 NMR spectrometer. The signal from H_2_O was suppressed using a 1 D NOESY pulse sequence (noesygppr1d). Samples were scanned thirty-two times with a relaxation delay of four seconds between each scan. The spectral width was set to 20.5 ppm.

Data were processed and analyzed in the manufacturer’s software (Topspin v.3.2). The raw spectra were treated with an exponential multiplication window function (line broadening of 0.3 Hz), before Fourier transformation. Spectra were calibrated to an external reference [4,4-dimethyl-4-silapentane-1-sulfonic acid (DSS) at 0.00 ppm].

### Statistics

The Shapiro–Wilk test of normality indicated that the mean P_o_, SF and MHC data were normally distributed, so the MHC-I data were compared between YA, MC, and HFP groups using a one-way ANOVA. MHC-IIA SF and MHC data were compared using *t* tests as there were only two groups (YAs and HFPs). MHC-IIA P_o_ data were not normally distributed so were compared using a Mann–Whitney test. P_o_ values normalized to MHC content, and the ratios of P_o_ measured in TES compared with imidazole solution were not normally distributed, so these data were compared between experimental groups using a Kruskal–Wallis test. A multiple *t* test was used for post hoc analysis of the TES:imidazole solution P_o_ ratios to compare groups using ranks from only the two groups being compared. P_o_ measured in the TES and imidazole dose–response experiments were compared with a one-way ANOVA with Tukey’s multiple comparison test. Paired *t* tests were used to compare data collected from the same fibers in two different solutions, (imidazole vs. TES solution, TES vs. imidazole substitution solution, imidazole vs. propionate substitution solution and TES solution vs. TES solution without glutathione). Data are presented as mean values with raw data points plotted, or mean ± SD.

## RESULTS

### Skinned Fiber Size and Force

A total of 316 fibers were studied across three experimental groups. Characteristics of the three groups are given in Table 1. Data from male and female subjects were grouped since numerous studies have found no effect of sex on human skinned fiber SF (18, 22, 23, 26, 27, 46–48). The distribution of fiber types, their CSAs and MHC content, are shown in Fig. 1. There were no differences between groups in MHC-I fiber CSA calculated using a circular (*P* = 0.8504) or elliptical (*P* = 0.6463) fiber shape. There were fewer MHC-IIA fibers from older participants, with smaller elliptical CSAs observed in fibers obtained from the HFPs compared with YAs (*P* = 0.0368) (Fig. 1*D*).

**Figure 2.**
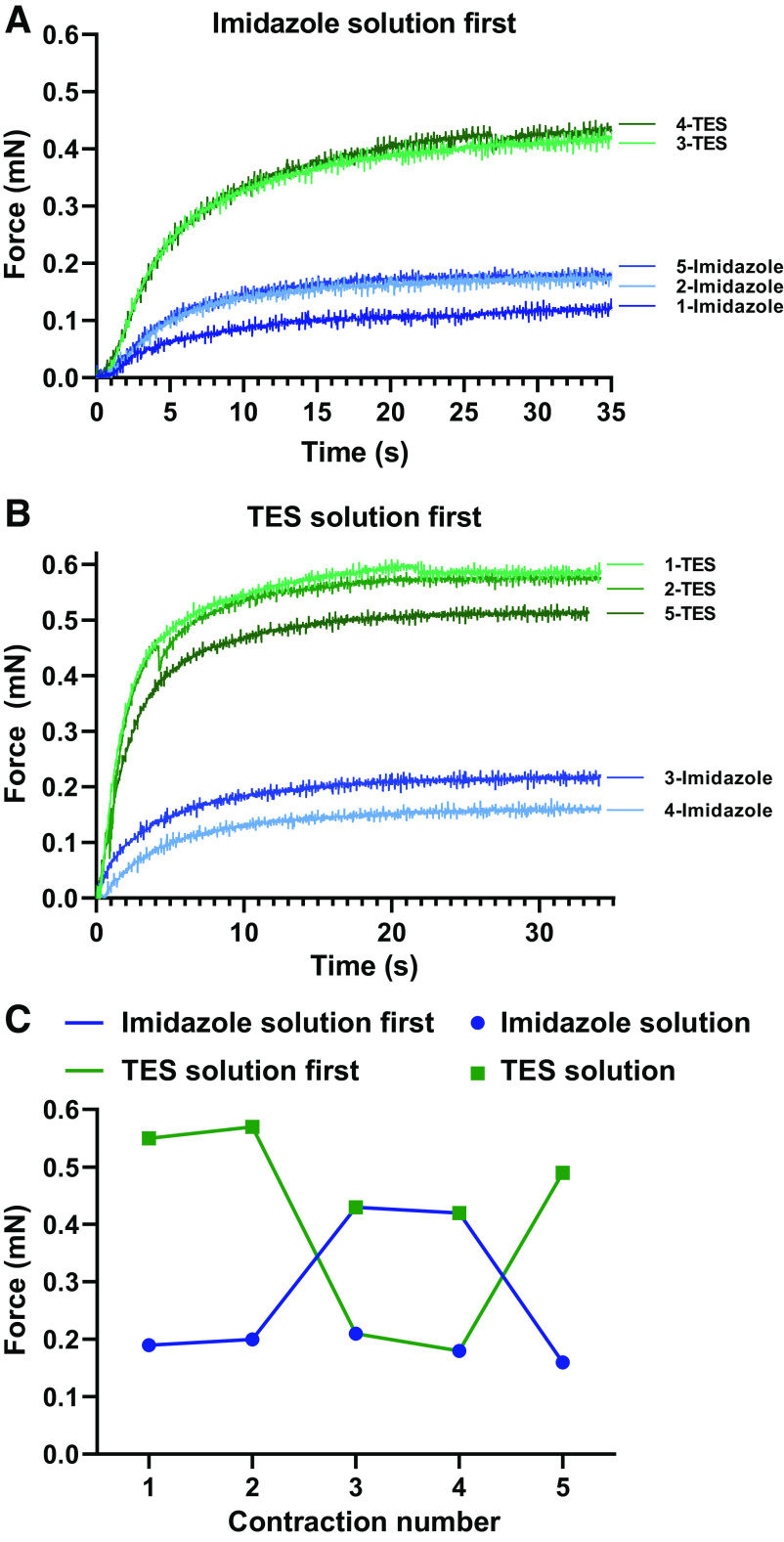
Outline of the five-contraction protocol and methodology. *A*: five representative force traces of an MHC-I skinned fiber from a young adult, contracting in imidazole solution first. *B*: as *A*, but contracting in TES solution first. *C*: the P_o_ data from *A* and *B*. The activating solution used first in the five-contraction sequence is indicated by the blue line (imidazole solution first) or green line (TES solution first) and whether a specific contraction occurred in imidazole (circles) or TES (squares) solutions is indicated by the different symbols. Mean force data from a given solution, used for subsequent calculation of SF, were taken as the mean of either contractions two and five, or three and four. MHC, myosin heavy chain.

To assess whether the chemical composition of activating solutions affects skinned fiber contraction force, peak isometric force (P_o_) was measured at 15°C in two solutions (Fig. 2), which differed in their pH buffers—an imidazole (20 mM) solution (31) and a TES (60 mM) solution (32). The imidazole solution is commonly used and was utilized in 30 of 61 of publications included in a recent meta-analysis (9). The full composition of the solutions used to prepare and activate single fibers are given in Tables 2 and 3.

To verify that P_o_ measured in a given activating solution was not affected by the order the two different solutions were used in, a five-contraction protocol was employed in all experiments (Fig. 2). Within imidazole solution or within TES solution, P_o_ measurements were found to be similar during the five-contraction protocol in YAs and MCs, irrespective of the order in which the solutions were used. However, in HFPs, the same consistency was not observed in MHC-I fibers, and P_o_ did not change when a different solution was used in MHC-IIA fibers (Supplemental Fig. S1).

**Figure 3.**
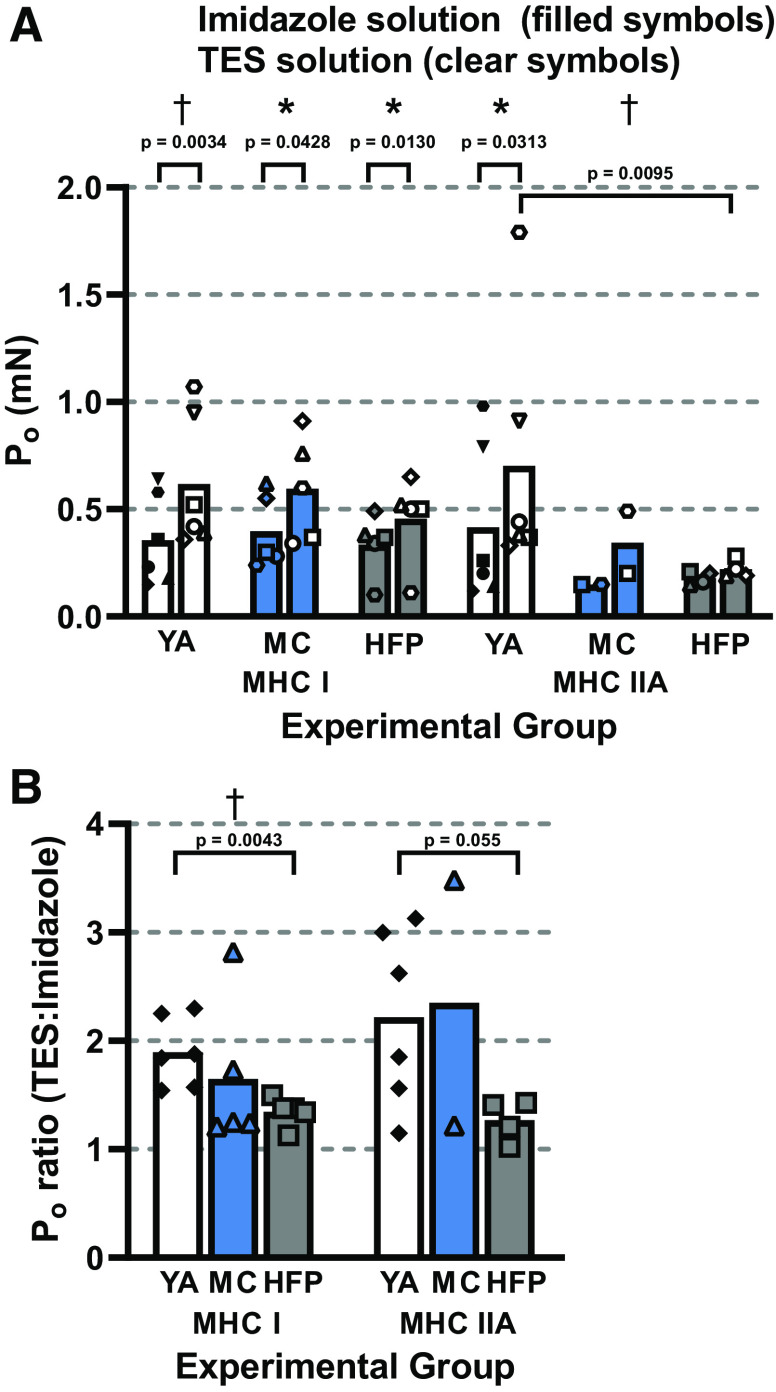
Effect of imidazole and TES activating solutions on skinned skeletal muscle fiber P_o_. Mean P_o_ in a given activating solution was calculated as the average of P_o_ from contractions 2 and 5, or 3 and 4, as described in the methods section. Individual symbols represent the mean value from different subjects. *A*: mean MHC-I P_o_ from YAs (*P* = 0.0034, *n* = 45), MCs (*P* = 0.0428, *n* = 72), and HFPs (*P* = 0.0130, *n* = 72) was significantly higher (*P* < 0.05) in TES solution compared with imidazole solution. The same significant effect was also observed in MHC-IIA fibers from YAs (*P* = 0.0313, *n* = 45) but not HFPs (*P* = 0.1102, *n* = 18). Only two MCs had more than two MHC-IIA fibers, so these data are plotted for comparative purposes but were not included in statistical analysis. One YA subject’s MHC-IIA fibers exhibited a strong response to TES solution compared with imidazole solution, but this value was less than two standard deviations from the mean so was not excluded as a statistical outlier. Between groups, P_o_ was significantly higher in YA than in HFP MHC-IIA fibers when studied in TES solution (*P* = 0.0095), but not imidazole solution (*P* = 0.6571). *B*: P_o_ increased significantly more (*P* = 0.0043) from imidazole solution to TES solution in MHC-I fibers from YAs (90%) than in HFPs (35%), but not MCs (65%). The same trend (*P* = 0.055) was observed in MHC-IIA fibers, as P_o_ increased by 121% in YAs and 27% in HFPs from imidazole solution → TES solution, but this trend was not statistically significant. MHC-I P_o_ data were compared between groups using a one-way ANOVA. MHC-IIA P_o_ was compared between YAs and HFPs using a Mann–Whitney test, as the data were not normally distributed. P_o_ data measured from the same fibers in imidazole solution compared with TES solution were compared using paired *t* tests for all fiber types and groups except YA MHC-IIA fibers, which were compared using a Wilcoxon test. The ratios of P_o_ measured in TES:imidazole solution were compared between groups using a Kruskal–Wallis test and post hoc analysis was conducted using a multiple *t* test to compare groups using ranks from only the two groups being compared. *P < 0.05, †*P* < 0.01. HFP, hip fracture patients; MC, master cyclists; MHC, myosin heavy chain; TES, N-tris(hydroxymethyl)methyl-2-aminoethanesulfonic acid pH buffer; YA, young adults.

Having verified that the P_o_ results were not affected by the order in which the activating solutions were used in the YA control group, P_o_ was then taken as the mean from two contractions in each activating solution, either the second and fifth or the third and fourth contractions. Within groups, MHC-I P_o_ was significantly greater in YAs (*P* = 0.0034), MCs (*P* = 0.0428), and HFPs (*P* = 0.0130) when measured in TES solution compared with imidazole solution. However, in MHC-IIA fibers, P_o_ was significantly increased in TES compared with imidazole solution in YAs only (*P* = 0.0313), and not in HFPs (*P* = 0.1102) (Fig. 3*A*). There were only two MCs with at least two MHC-IIA fibers, preventing statistical comparison.

When studied as a percentage change (Fig. 3*B*), the increase in P_o_ from the imidazole to TES solution in MHC-I fibers was 90% (32% SD) in the YAs. This was significantly (*P* = 0.0043) greater than the 35% (13.5% SD) increase in the HFPs and indicates that the TES solution had a more potent effect on the YA than HFP fibers. The 65% (69% SD) change in MC fibers lay between these values and was not significantly different to the other two groups. For the MHC-IIA fibers, there was a tendency (*P* = 0.055) for a greater percentage increase in P_o_ from the imidazole to the TES solution, which increased by 121% (81% SD) in YAs compared with 27% (19% SD) in the HFPs. This may be because there is one less MHC-IIA data point in the HFP group, since one participant only had a single MHC-IIA fiber and was therefore excluded from this analysis.

Finally, when experimental groups were compared with each other the mean P_o_ of MHC-I fibers was similar in both activating solutions across all groups. MHC-IIA fiber P_o_ was also similar between groups when measured in the imidazole solution, but a significant (*P* = 0.0095) difference was observed between YAs and HFPs when P_o_ data from TES solution were compared (Fig. 3*A*).

### Specific Force

**Figure 4.**
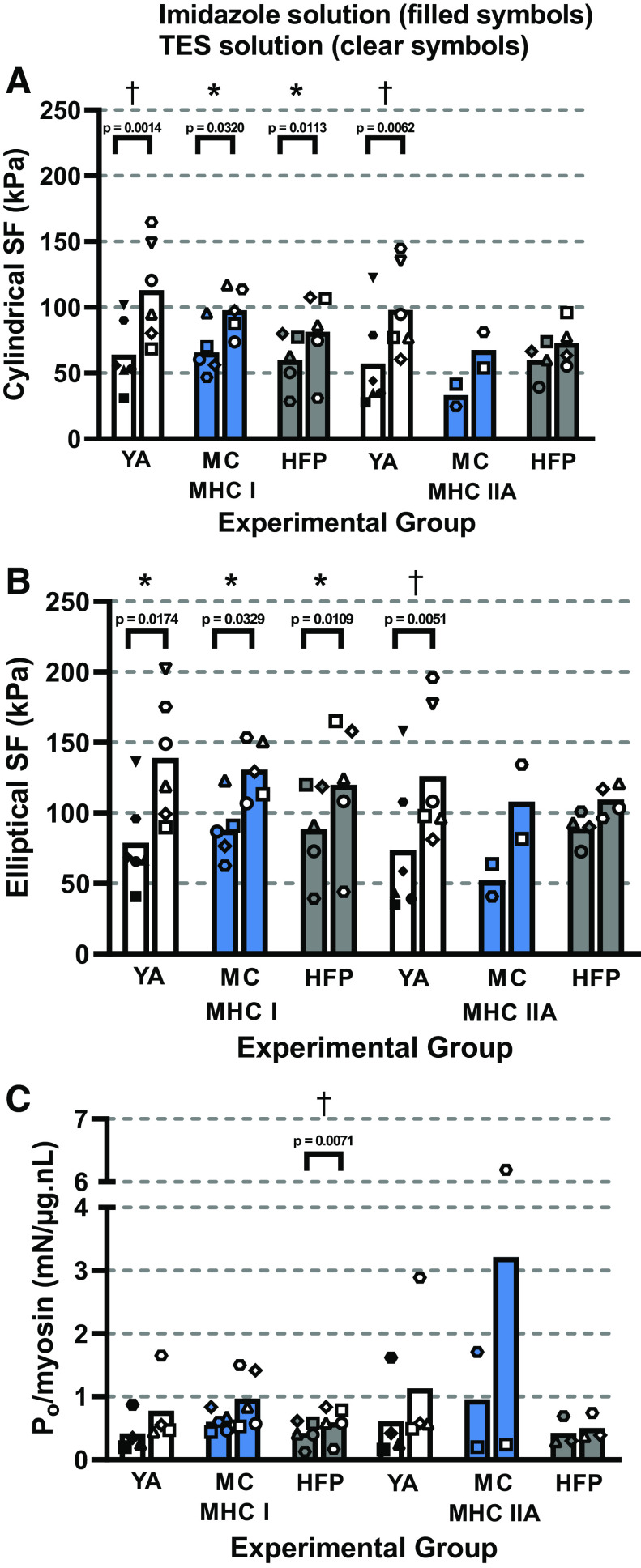
Comparison of mean cylindrical SF, elliptical SF, and P_o_/MHC measured in imidazole solution compared with TES solution. MHC-I cylindrical SF (*A*) values were significantly higher when measured in TES solution compared with imidazole solution in YAs (*P* = 0.0014, *n* = 45), MCs (*P* = 0.0320, *n* = 72), and HFPs (*P* = 0.0113, *n* = 72). MHC-I elliptical SF (*B*) values from YAs (*P* = 0.0174), MCs (*P* = 0.0329), and HFPs (*P* = 0.0109) were also higher in TES compared with imidazole solution. The same significant effect was also observed in MHC-IIA fibers from YAs (*n* = 45) for cylindrical (*P* = 0.0062) and elliptical (*P* = 0.0051) SF. P_o_/MHC (*C*) was significantly higher (*P* = 0.0071) in TES solution in MHC-I fibers from HFPs (*n* = 67), and not YA (*n* = 24) or MCs (*n* = 68), or MHC-IIA fibers from each group (YA *n* = 20; MC *n* = 5; HFP *n* = 12). Individual symbols represent the mean value from different subjects. MHC-I SF was compared between groups using a one-way ANOVA, and YA and HFP MHC-IIA SF was compared using an unpaired *t* test. SF measured in imidazole or TES solution was compared within groups using a paired *t* test for both MHC-I and -IIA fibers. MHC-I P_o_/MHC values were compared between groups using a Kruskal–Wallis test and MHC-IIA P_o_/MHC values using a Mann–Whitney test. MHC-I and -IIA P_o_/MHC values measured in imidazole or TES solution were compared within YAs using a Wilcoxon test. MHC-I MC and HFP P_o_/MHC values were compared using a paired *t* test. MHC-IIA HFP P_o_/MHC values were compared using a Wilcoxon test. *P < 0.05, †*P* < 0.01. HFP, hip fracture patients; MC, master cyclists; MHC, myosin heavy chain; TES, N-tris(hydroxymethyl)methyl-2-aminoethanesulfonic acid pH buffer; YA, young adults.

SF was calculated by normalizing P_o_ to fiber CSA assuming either a cylindrical (Fig. 4*A*) or an elliptical CSA (Fig. 4*B*) to determine whether CSA shape affected the comparison of SF between young and elderly groups. P_o_ was also normalized to MHC content (Fig. 4*C*). Within groups, MHC-I cylindrical SF was higher when fibers were activated in TES compared with imidazole solution in YAs (*P* = 0.0014), MCs (*P* = 0.0320) and HFPs (*P* = 0.0113). MHC-I elliptical SF was also higher in TES solution in YAs (*P* = 0.0174), MCs (*P* = 0.0329) and HFPs (*P* = 0.0109) (Fig. 4, *A* and *B*). MHC-IIA fiber cylindrical SF (*P* = 0.0062) and elliptical SF (*P* = 0.0051) were significantly higher in TES solution compared with imidazole solution in YAs, but not HFPs. Between groups, no significant difference in MHC-I or -IIA cylindrical SF (Fig. 4*A*) or elliptical SF (Fig. 4*B*) was observed, when measured in either TES or imidazole solutions. This was despite the significantly higher MHC-IIA P_o_ in TES solution in YAs compared with HFPs and was accounted for by the lower MHC-IIA fiber CSAs in the HFPs. P_o_ normalized to MHC content was increased significantly (*P* = 0.0071) in TES solution compared with imidazole solution in MHC-I fibers within the HFP group, but this was not observed in other groups or in the MHC-IIA fibers studied (Fig. 4*C*). The large variability, typical of skinned fiber experiments, may be the cause of the statistically negative result of P_o_ normalized to myosin compared with CSA (Supplemental Fig. S5).

### The Impact of TES and Imidazole on Skinned Fiber-Specific Force

**Figure 5.**
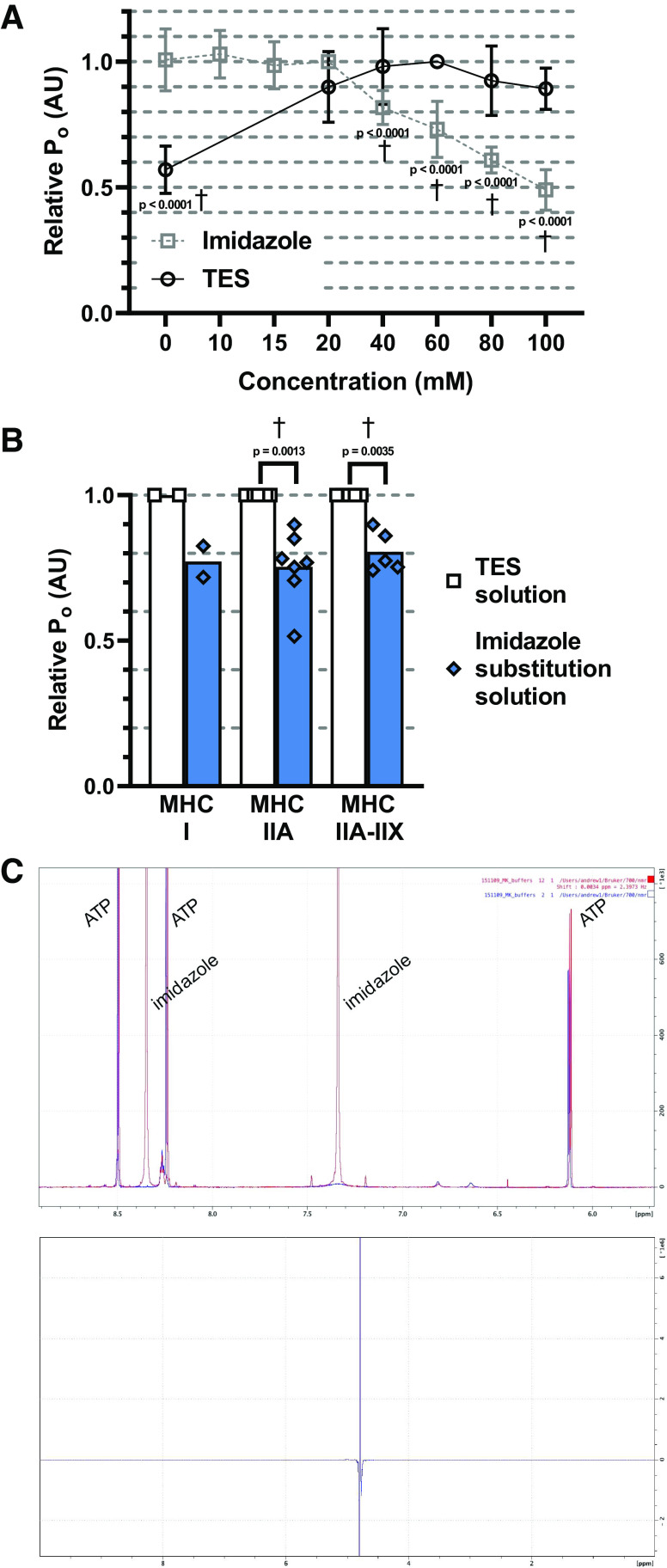
The effect of TES or imidazole pH buffers on the skinned fiber P_o_ response. *A*: skinned fibers were activated in solutions, which contained either TES (○) or imidazole (□) pH buffers at different concentrations. Data are presented in order of increasing buffer concentration on the *x*-axis. Data are mean values normalized to the peak P_o_ observed ± SD. P_o_ was significantly (*P* < 0.0001) lower in a solution containing 0 mM TES compared with a solution containing an optimal amount of TES (60 mM) (*n* = 8). In contrast, no significant difference (*P* > 0.05) in P_o_ was observed when fibers contracted in solutions containing between 20 and 100 mM of TES (*n* = 9). The imidazole dose–response curve was carried out in two separate experiments, the first between 0 and 20 mM (*n* = 12) and the second between 20 and 100 mM (*n* = 9) of imidazole. P_o_ was plotted relative to force at 20 mM of imidazole, the concentration most commonly used in the literature. 40, 60, 80, and 100 mM of imidazole had a significantly (*P* < 0.0001) negative impact on P_o_. *B*: the mean P_o_ of the same individual fibers activated in TES solution (60 mM TES) vs. imidazole substitution solution (20 mM imidazole). P_o_ was significantly (*P* < 0.05) lower in the same fibers contracting in imidazole substitution solution compared with TES solution in MHC-IIA (*P* = 0.0013, *n* = 7) and MHC-IIA–IIX fibers (*P* = 0.0035, *n* = 5). P_o_ from MHC-I fibers in the imidazole substitution solution was lower than TES solution but was not statistically tested due to an *n* = 2. *C*: a portion of the ^1^H NMR spectra illustrating the compounds detected in TES or imidazole substitution solution. There was no indication of reaction products as a result of the use of either TES or imidazole. The only compounds, which were found to differ, were those expected, for example, the two large peaks corresponding to imidazole (pictured) which were in imidazole substitution solution (red peaks) but not TES solution (blue peaks). The blank spectrum revealed the presence of H_2_O only. P_o_ measured in the same fibers at different concentrations of imidazole or TES were compared using a one-way ANOVA with Tukey’s multiple comparison test. P_o_ data measured in TES solution vs. the same solution, which excluded TES, or vs. imidazole substitution solution were compared using a paired *t* test. †*P* < 0.01. ^1^H NMR, proton nuclear magnetic resonance spectroscopy; MHC, myosin heavy chain; TES,N-tris(hydroxymethyl)methyl-2-aminoethanesulfonic acid pH buffer.

To determine the chemical cause of the different contractile responses elicited by imidazole and TES solutions, three chemical differences between the solutions were tested using skinned fibers from a YA individual: *1*) the pH buffer used, *2*) the major anion, and *3*) the use of a reducing agent. As these data were collected using skinned fibers from one YA subject they must be interpreted with caution. A dose-response curve was plotted to determine the optimal concentration of the pH buffering compound for skinned fiber force production. P_o_ was significantly decreased in the absence of TES (*P* < 0.0001), whereas no difference was observed between all other concentrations tested (Fig. 5*A*). In activating solutions containing imidazole, no significant differences in P_o_ were observed between 0 and 20 mM. However, compared with 20 mM, P_o_ was significantly (*P* < 0.0001) reduced at all higher concentrations of imidazole (Fig. 5*A*).

Once the optimal concentrations of imidazole (20 mM) and TES (60 mM) were determined (which corresponded to the concentrations used in the experiments on the young and older fibers reported above in Figs. 2, 3, and 4), a direct comparison of how the pH buffers affect skinned fiber force production was made. It was necessary to control for differences in the anion (KCl vs. K-propionate) and reducing agent (glutathione) used in the imidazole compared with the TES solution. Therefore, a new activating solution, “imidazole substitution solution” (Table 3), was made which was chemically identical to the TES solution but in which 60 mM TES was replaced with 20 mM imidazole. Skinned fiber P_o_ in imidazole substitution solution was significantly (*P* < 0.05) and consistently (Supplemental Fig. S3) lower than in TES solution for all MHC isoforms studied (Fig. 5*B*), indicating that the incorporation of TES augmented skinned fiber P_o_.

^1^H NMR analysis was undertaken for each activating solution to identify any chemical changes, which might explain the mechanism causing the force augmenting effect of TES compared with imidazole pH buffer. However, ^1^H NMR spectra of TES and imidazole substitution solutions corresponded to those expected as the sum of spectra of the individual components (Table 3). There was no indication of chemical modifications to the compounds in either TES or imidazole substitution solutions. The distilled water sample was shown to be pure, excluding the presence of any compounds other than H_2_O at detectable concentrations (Fig. 5*C*).

The major anion in the TES solution was K-propionate, and KCl in the imidazole solution. A new solution was made which was similar to the imidazole solution but with K-propionate substituted in for KCl. MHC-I fiber force was similar (*P* = 0.0997) in both solutions, although MHC-IIA fiber force was lower (*P* = 0.0011) in K-propionate containing solution (Supplemental Fig. S6*B*). Finally, TES solution contained the reducing agent glutathione whereas imidazole solution did not. TES solution was compared with a chemically identical solution which did not contain glutathione and no effect on P_o_ was observed in MHC-I (*P* = 0.1764) or MHC-IIA (*P* = 0.2656) fibers (Supplemental Fig. S6*A*).

## DISCUSSION

The present study examined SF in single human skinned skeletal muscle fibers from young and older individuals and aimed to investigate the effects of two factors that may have influenced the conclusions drawn from previous research. The first is the type of individual from whom muscle fibers are obtained, which has traditionally ranged from frail to very healthy and physically active older individuals. We therefore studied fibers from two groups of older people: those undergoing hip surgery (with comorbidities) and healthy, active master cyclists. This represented two extremes of older phenotypes. Indeed, D’Antona et al. (49) showed an activity dose-response relationship in SF in single fibers obtained from older individuals ranging from “immobilized” through to master runners. The second factor is related to methodological considerations when performing experiments on single-skinned fibers: the solution used to activate fibers and the way in which fiber force is normalized. Our recent systematic review (9) suggested that much of the difference in SF between healthy young adults that was apparent in studies from different research groups was likely explained by differences in the composition of the solutions used to activate permeabilized fibers.

By obtaining fibers from more defined older individuals, and by exposing fibers to more than one activating solution, while using a range of different normalization approaches, we aimed to gain further insight into the effects of age and activity on muscle contractile quality.

The main findings were that within all groups, P_o_, elliptical SF, and cylindrical SF were significantly (*P* < 0.05) higher in fibers activated using TES than imidazole solution in MHC-I fibers. However, in MHC-IIA fibers, this was only the case for YAs and not for HFPs. There were too few MC MHC-IIA fibers for statistical comparison. Furthermore, the percentage increase in MHC-I force when moving between the two solutions was significantly greater in YAs than HFPs, with a strong tendency also observed in MHC-IIA fibers. Together, these results suggest an alteration in the behavior of force-generating processes in fibers of HFPs, which would not have been revealed using a single activating solution approach.

### P_o_ Measured in Different Activating Solutions

A novel finding of the present study is that YA skinned fibers were able to respond to a change in chemical environment (TES solution) more than HFPs but not MCs. This was indicated by the greater (*P* < 0.05) increase in YA MHC-I P_o_ between imidazole → TES solution. Also, MHC-IIA P_o_ was similar between YAs and HFPs in imidazole solution, but due to the greater response of YA fibers to TES solution MHC-IIA P_o_ became significantly greater than in HFPs (Fig. 3).

We identified the pH buffer used to be the primary chemical cause of the augmentative effect of TES solution on force. In skinned fiber experiments, the control of pH in the activating solution is necessary due to processes such as the hydrolysis of ATP during myosin attachment or detachment to actin, which releases H^+^ (50). When compared at their optimal concentrations and controlling for differences in the anion and reducing agent used, skinned fiber SF was significantly (*P* < 0.05) and consistently higher in solution containing 60 mM TES compared with 20 mM imidazole (imidazole substitution solution) for all MHC isoforms examined (Fig. 5*B*). To the best of our knowledge, this is the first time an effect of pH buffer on P_o_ has been demonstrated at the same pH. Previous studies found no difference in skinned fiber P_o_ between solutions containing imidazole, Tris(hydroxymethyl)aminomethane pH buffer (Tris), and Tris(hydroxymethyl)aminomethane pH buffer (Bis-Tris) (51) or containing TES, (2-(N-morpholino)ethanesulphonic acid) pH buffer (MES), and (N,N-bis(2-hydroxyethyl)-2-aminoethanesulphonic acid) pH buffer (BES) (52). Given the present study’s finding, this suggests that TES, MES, and BES may elicit higher forces than imidazole, Tris, and Bis-Tris, but further experiments are required to test this. The fact that the majority of human-skinned fiber experiments do not use TES suggests that submaximal isometric forces may be being reported. Furthermore, different pH buffer concentrations can affect the binding constant of calcium to EGTA (53) suggesting that the use of different pH buffers at different concentrations may affect other measurements such as the calcium sensitivity of force.

The mechanism of the augmentative effect of TES on P_o_ was investigated using ^1^H NMR analysis, which revealed that new reaction products were not formed as a result of TES or imidazole pH buffer so do not explain the effect on P_o_ (Fig. 5). ^1^H NMR analysis cannot detect non-H^+^ containing metal ions such as Mg^2+^, which can compete with Ca^2+^ for binding sites on the myofilaments (54, 55) so does not rule out that TES augments P_o_ by reducing competition for Ca^2+^ binding on troponin. The inclusion of TES in activating solution likely caused a change in skinned fiber cross-bridge behavior, since MHC content was similar between YA and HFPs in both MHC-I and -IIA fibers, suggesting a similar number of available cross bridges. Previous studies have reported that differences in skinned fiber stiffness, i.e., the number of bound cross bridges, can explain SF differences despite similar MHC content (36, 56). Therefore, the greater response to TES solution of YA fibers may be due to increased stiffness relative to HFP fibers, although further experiments are required to test this. Alternatively, reduced SF in older men (12) and rats (57) has been attributed to more cross bridges in a low force-producing state. However, P_o_ per unit MHC was similar between YAs and HFPs, suggesting that TES did not cause YAs to produce more force per cross bridge than HFPs.

Lower skinned fiber SF despite similar MHC content has also been associated with posttranslational modifications such as phosphorylation of contractile proteins and oxidation of MHC, contributing to lower SF in older compared with young participants (58). Therefore, the smaller capacity for force generation in HFP fibers may also be related to oxidative modifications affecting cross-bridge function. Furthermore, the effect of the pH buffers on force production may be due to modifications of the contractile proteins, which are differentially modulated in YAs compared with HFPs.

### Comparison of MHC Content and SF across Different Experimental Groups

Different approaches have been taken to normalize P_o_ to CSA, which include the use of a circular or elliptical shape as well as assumptions regarding fiber swelling in this permeabilized preparation (9). We found that normalization to either shape had no effect in between-group comparisons.

Studies that have measured both skinned fiber MHC protein content and SF from both young and older human cohorts have typically reported that SF is positively related to MHC protein content (15, 22, 36, 56, 59, 60). D’Antona et al. (49) reported that skinned fiber SF was lower in immobilized but not physically active elderly individuals compared with young controls and had previously (60) found that lower skinned fiber SF in elderly cohorts was due to lower skinned fiber MHC content. Therefore, the present study’s finding that similar skinned fiber SF between groups was associated with similar MHC content is mechanistically consistent with previous work (Fig. 1*F*) (15, 22, 60).

Given the link between skinned fiber MHC content and SF, an interesting question is why all older cohorts studied have not exhibited reduced MHC content? Physical inactivity such as cast immobilization (15, 60) or long-term (>35 days) bed rest (59, 61) is associated with reduced MHC content whereas physical activity such as resistance training increases skinned fiber MHC content and SF (15). The fact that the YA or MC group did not exhibit higher MHC content than HFPs may indicate that the physical activity level of HFPs was not low enough to elicit a decrease in skinned fiber MHC content. Alternatively, the physical activity level required to alter MHC content and subsequently skeletal muscle function may differ between individuals (62).

Another factor, which may affect single fiber MHC content, is diet. A nutritional intervention without exercise preserved human soleus and VL skinned fiber SF despite long-term bed rest (63, 64), and young and older cohorts with similar nutritional intake had similar VL MHC-I fiber SF (65). Consistent with this, well-nourished but physically inactive sled dogs did not exhibit myofilament depletion, whereas undernourished and inactive sled dogs did (66). Furthermore, overexercised but malnourished anorexia nervosa patients exhibited separation and segmental loss of myofibrils (67). These findings suggest that adequate nutritional intake may preserve both muscle protein content and skinned fiber SF in the absence of exercise. Diet was not a variable accounted for in the present study or in the majority of other studies investigating skinned fiber SF in aging but may be an important consideration for future research.

### Implications of Using Different Activating Solutions

The difference in force between TES and imidazole-activating solutions has several implications. The large variation in published human-skinned fiber SF values (9) and shown experimentally in the same laboratory here, is likely to result from the different activating solutions used by different research groups. This makes comparing and interpreting SF differences between studies complicated and suggests that those that have used imidazole have underestimated maximal force generation, although imidazole did not negatively affect P_o_ at the concentration used (20 mM). Furthermore, MHC-IIA P_o_ was significantly greater in YAs compared with HFPs when measured in TES but not imidazole solution. This suggests that conclusions regarding skinned skeletal muscle fiber physiology may be different depending on the activating solution used, although when P_o_ was normalized to CSA no differences in SF were observed. Testing whether the dose-response to a given compound is the same in samples from young and elderly groups could indicate whether different solutions elicit maximal force from the different groups. This would be a potential area for further research. Finally, if low force production is an exclusion criterion, some low force-producing fibers in imidazole solution may be excluded from analysis, whereas they could be included if studied in TES solution. While TES solution was found to be better than imidazole solution for eliciting a maximal contraction, further experiments would be required to verify that TES solution is “optimal.”

### Summary and Conclusions

The present study has demonstrated that chemically skinned human fibers are highly sensitive to their chemical environment and that using TES as opposed to imidazole as the pH buffer results in higher forces. Our study reports similar MHC-I and MHC-IIA SF values (irrespective of the mode of CSA determination) between all groups. This aligns with the similar MHC content in fibers from the different groups. However, despite similar SF and MHC content, disparities in the sensitivity of fibers to activation in solutions containing different pH buffers suggests there may be differences in cross-bridge behavior between older individuals from distinct activity spectra and young adults. This may require a combination of approaches, including using different activating solutions to reveal underlying mechanisms. The study also highlights the need for an optimal activating solution to be developed, which might be used across different research groups.

## DATA AVAILABILITY

Data will be made available upon reasonable request.

## SUPPLEMENTAL DATA

10.6084/m9.figshare.21512427 Supplemental Tables S1–S9: https://doi.org/10.6084/m9.figshare.21512427.

## GRANTS

This work was funded by a King’s College London Health schools studentship.

## DISCLOSURES 

No conflicts of interest, financial or otherwise, are declared by the authors.

## AUTHOR CONTRIBUTIONS

M.K., R.D.P., N.R.L., M.G., O.B., R.C.W., J.O., and S.D.R.H. conceived and designed research; M.K. and R.A.A. performed experiments; M.K., R.A.A., and R.C.W. analyzed data; M.K., R.A.A., R.C.W., J.O., and S.D.R.H. interpreted results of experiments; M.K. prepared figures; M.K., J.O., and S.D.R.H. drafted manuscript; M.K., R.D.P., N.R.L., R.A.A., M.G., O.B., J.O., and S.D.R.H. edited and revised manuscript; M.K., R.D.P., N.R.L., R.A.A., M.G., O.B., J.O., and S.D.R.H. approved final version of manuscript.
